# Rapid acid treatment of *Escherichia coli*: transcriptomic response and recovery

**DOI:** 10.1186/1471-2180-8-37

**Published:** 2008-02-26

**Authors:** Geetha Kannan, Jessica C Wilks, Devon M Fitzgerald, Brian D Jones, Sandra S BonDurant, Joan L Slonczewski

**Affiliations:** 1 Department of Biology, Kenyon College, Gambier, OH, 43022 USA; 2 Department of Mathematics, Kenyon College, Gambier, OH, 43022 USA; 3 Gene Expression Center, University of Wisconsin, Madison, WI 53706, USA

## Abstract

**Background:**

Many *E. coli *genes show pH-dependent expression during logarithmic growth in acid (pH 5–6) or in base (pH 8–9). The effect of rapid pH change, however, has rarely been tested. Rapid acid treatment could distinguish between genes responding to external pH, and genes responding to cytoplasmic acidification, which occurs transiently following rapid external acidification. It could reveal previously unknown acid-stress genes whose effects are transient, as well as show which acid-stress genes have a delayed response.

**Results:**

Microarray hybridization was employed to observe the global gene expression of *E. coli *K-12 W3110 following rapid acidification of the external medium, from pH 7.6 to pH 5.5. Fluorimetric observation of pH-dependent tetR-YFP showed that rapid external acidification led to a half-unit drop in cytoplasmic pH (from pH 7.6 to pH 6.4) which began to recover within 20 s. Following acid treatment, 630 genes were up-regulated and 586 genes were down-regulated. Up-regulated genes included amino-acid decarboxylases (*cadA, adiY*, *gadA*), succinate dehydrogenase (*sdhABCD*), biofilm-associated genes (*bdm*, *gatAB*, and *ymgABC*), and the Gad, Fur and Rcs regulons. Genes with response patterns consistent with cytoplasmic acid stress were revealed by addition of benzoate, a membrane-permeant acid that permanently depresses cytoplasmic pH without affecting external pH. Several genes (*yagU*, *ygiN*, *yjeI*, and *yneI*) were up-regulated specifically by external acidification, while other genes (*fimB*, *ygaC*, *yhcN*, *yhjX*, *ymgABC*, *yodA*) presented a benzoate response consistent with cytoplasmic pH stress. Other genes (the *nuo *operon for NADH dehydrogenase I, and the HslUV protease) showed delayed up-regulation by acid, with expression rising by 10 min following the acid shift.

**Conclusion:**

Transcriptomic profiling of *E. coli *K-12 distinguished three different classes of change in gene expression following rapid acid treatment: up-regulation with or without recovery, and delayed response to acid. For eight genes showing acid response and recovery (*fimB*, *ygaC*, *yhcN*, *yhjX*, *ymgABC*, *yodA*), responses to the permeant acid benzoate revealed expression patterns consistent with sensing of cytoplasmic pH. The delayed acid response of *nuo *genes shows that NADH dehydrogenase I is probably induced as a secondary result of acid-associated metabolism, not as a direct response to cytoplasmic acidification.

## Background

Extreme-acid survival is an important virulence factor for human pathogens such as *E. coli *strain O157:H7 [1]. In order to colonize the gastrointestinal tract, *Escherichia coli *and other enteric bacteria must be able to grow in environments at extreme pH such as the duodenum (pH 9–10) and the stomach (pH 2–4) [2-4]. In the gastrointestinal tract, enteric bacteria are subjected to acid stress from strong acid (HCl) as well as bacterial fermentation products such as acetic, propionic, and butyric acids, which are membrane-permeant weak acids [5]. *E. coli *preserves the integrity of proteins and nucleic acids present in the cytoplasm by maintaining cytoplasmic pH at approximately pH 7.6, over a wide range of external pH [6,7]. Low pH up-regulates genes required for survival under more extreme acid conditions, such as the arginine- and glutamate-dependent acid resistance systems [8-10].

In *E. coli *K-12, many studies show that adaptation to low or high pH stress involves regulation of gene expression and protein synthesis, as well as post-translational and regulation of protein function [11-14]. For example, acid induces the lysine decarboxylase operon *cadBA *under control of the signal-transducing regulator CadC [13,14]. Lysine decarboxylase (CadA) removes CO_2 _from lysine, releasing cadaverine, a base that counteracts acidity. Other kinds of genes up-regulated by acid include periplasmic chaperones, inner-membrane and outer-membrane proteins, acetate-stress proteins, and systems utilizing the proton gradient, such as motility and chemotaxis [11]. Base-induced proteins include fermentation pathways generating acidic products, and metabolic complexes that import protons or minimize proton export, such as the F_1_F_o _ATPase and cytochrome *d *oxidoreductase (*cydAB*) [11,12].

While gene expression as a function of steady-state external pH has received extensive study, less is known about the dynamic response of *E. coli *to a sudden pH change. Rapid acidification (within 10 s) of the external medium causes the cytoplasmic pH to fall, followed by recovery within minutes to close to the original value [15,16]. For the *cadBA *operon, expression is upregulated within 4 min of rapid acidification, and downregulated within 4 min after pH neutralization [17]. But other genes showing pH-dependent expression ratios under growth for several generations at different pH values may be dependent on secondary effects of growth with pH stress. The gene products required to recover from rapid change in external pH (with a transient failure of pH homeostasis) may differ substantially from those elevated or repressed during steady-state growth at low or high pH, where pH homeostasis is maintained. Furthermore, few studies distinguish the effects of cytoplasmic versus external pH on gene expression; for example, *yfiD *expression responds to membrane-permeant weak acids that depress cytoplasmic pH without affecting external pH [18].

In this study we used microarray hybridization to investigate the transcriptomic response to a sudden acid shift. This approach enabled us to distinguish between genes up-regulated immediately upon acid shift, versus those whose expression increases after some time in the presence of acid. Genes of interest were followed up with quantitative real-time PCR (qPCR) using a membrane-permeant weak acid to assess whether the effect of acid treatment could be associated with the decrease of cytoplasmic pH.

## Results and Discussion

### Cytoplasmic pH change

The effect of external acidification on the *E. coli *cytoplasmic pH under our experimental conditions was tested, in order to assess the state of the cytoplasm during our transcriptional profiling. The probe used was pH-dependent fluorescence of *tetR*-YFP [16].

**Figure 1:**
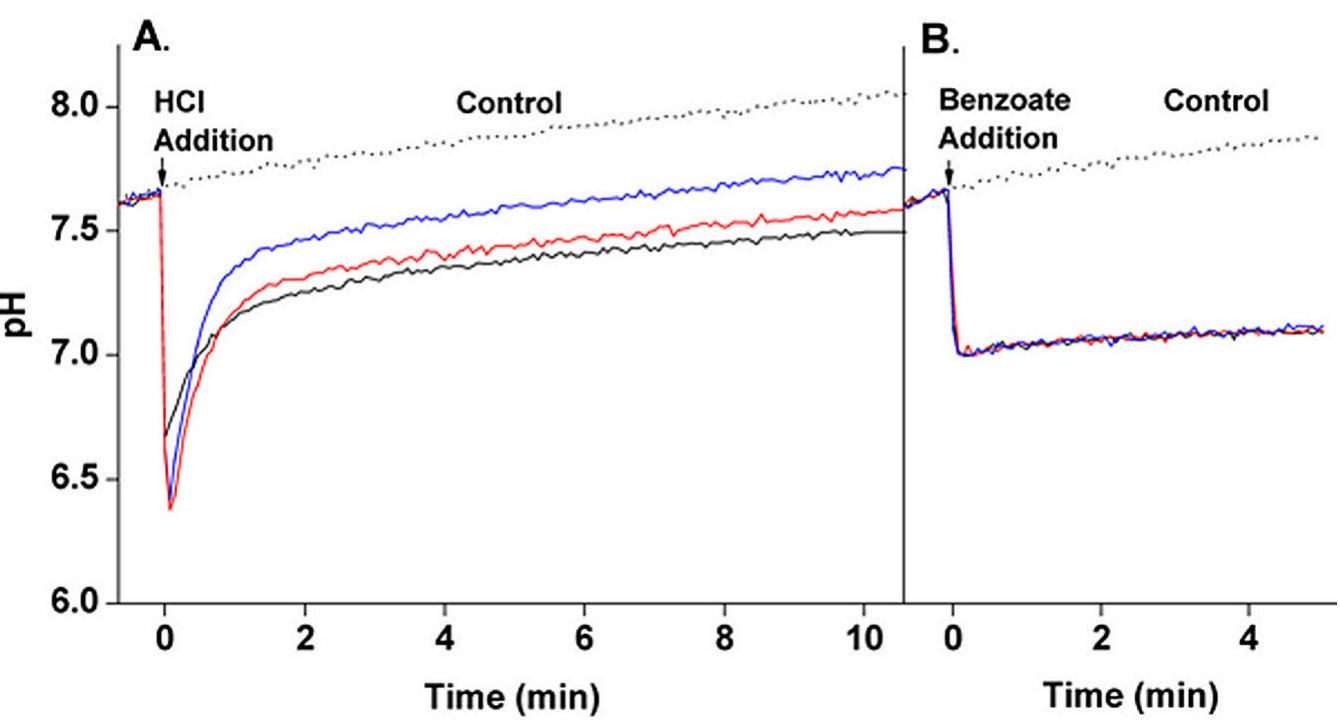
**Effects of external acid shift and benzoate addition on cytoplasmic pH**. (A) Cultures were suspended at pH 7.6 in supplemented M63 minimal media (20 mM HOMOPIPES). At time zero, 8.5 mM HCl was added to shift the external pH from pH 7.6 to 5.5. For the control, KCl (8.5 mM at pH 7.6) was added instead of HCl (B) The permeant weak acid sodium benzoate (30 mM) was added at time zero to cultures suspended at pH 7.0 in supplemented M63 minimal meida (50 mM HOMOPIPES). As a control, HOMOPIPES (pH 50 mM at 7.0) was added instead of sodium benzoate. For both experimental conditions, fluorescence was converted to pH units using the standard curves as described in Materials and Methods. Under each condition, three independent cultures were tested.

The external pH was acidified by adding HCl (8.5 mM) to cell cultures of *E. coli *W3110 containing a *tetR*-YFP plasmid, suspended in media adjusted to pH 7.6. Upon HCl addition, the cytoplasmic pH fell within 5 s to approximately pH 6.4 (Fig. 1A). Recovery began approximately 4 s after the cytoplasmic pH reached the lowest point (~pH 6.4). After 10 min, the cytoplasmic pH ranged between pH 7.5 and pH 7.7. Each replicate culture exhibited a biphasic recovery, which is consistent with the recovery pattern previously described by ref [16]. As a control, KCl was added instead of HCl (8.5 mM KCl, pH 7.6) in order to detect changes in fluorescence signal that were independent of pH change. Upon KCl addition, there was no significant change in fluorescence signal.

For comparison, the effect of a permeant-acid stress was measured under our experimental conditions [16]. Addition of a permeant acid such as benzoate at high concentration depresses cytoplasmic pH with little or no recovery, without affecting external pH [15]. Benzoate (30 mM) was added to cell cultures of W3110 containing p-*tetR*-YFP, which were suspended in media pH adjusted to pH 7.0. After addition of 30 mM benzoate, the cytoplasmic pH fell within 8 s to that of the external medium (pH 7.0, Fig. 1B). Over the period of 5 min during which spectra were recorded, the cytoplasmic pH made a minimal recovery. (A period of only 5 min is shown because the YFP protein begins to destabilize in the presence of benzoate.) Buffer (50 mM HOMOPIPES, pH 7.0) was added as a control, and did not cause a significant change in fluorescence signal.

### RNA hybridization following rapid acidification

For acid treatment and RNA hybridization, *E. coli *strain W3110 was cultured to early log phase (OD_600 _= 0.2) at pH 7.6 (+/- 0.2 pH units). pH_ext _7.6 is equivalent to the cytoplasmic pH, and thus cells suspended at pH_ext _7.6 exhibit minimal pH regulated gene expression relative to rapidly acidified cultures. At time zero (OD_600 _0.2), HCl was added rapidly, resulting in the acidification of the external medium to pH 5.5. At time points 0, 1, 5, and 10 min, 10 mL of culture were removed. The pH of the media remained at pH 5.5 (+/- 0.2 pH units) after the 10 min sample was taken.

At time points 0, 1, 5, and 10 min, RNA was purified and cDNA generated as described under Methods. The cDNA from five independent cultures of each time-point were hybridized to Affymetrix antisense *E. coli *arrays. Model-based expression indices were calculated from the probe-level data from Affymetrix DAT files using dChip software [19,12]. Array data were deposited at the NCBI Gene Expression Omnibus (accession GSE4778).

### Time-dependent variation

To determine the effect of time on differential gene expression, the log_2 _transforms of normalized model-based expression values from the microarray probe level data were analyzed during the 10-min interval. A mixed-effects linear model for longitudinal data was applied using the software SAS Mixed Procedure (Proc Mixed). For each gene, the mixed-effects model was applied at a significance level of 0.001 to determine whether or not the gene expression profile deviates significantly from a flat line over time, which indicates no time effect. The model takes into account time (fixed effect), the random deviation among biological replicates (random effects), and random error to determine significant time-dependence for gene variation [20-22].

For array hybridization, our experimental design and analysis were consistent with the "consensus" recommendations of Allison [23] in that we included an ample number of biological replicates (five independent cultures for each time point), assuring a low false-positive rate (0.001, or one false-positive in 1,000 genes) as well as a high power of detection. The power of detection of changes in gene expression over time was estimated based on the observed biological variation among flask cultures (90% of the genes showed a standard deviation of less then 0.08) and the technical error term (90% of the genes showed a standard deviation of less than 0.28). The power of detection was estimated for two cases of expression change using a Monte Carlo simulation based on 5,000 trials. For a gene whose expression increases two-fold over 10 min, the power of detection was 96%. For a gene whose expression increases two-fold by 5 min, with partial recovery by 10 min, the power of detection was 97%.

Of the 4,377 genes on the array, 1,582 (36% of the *E. coli *genome) showed significant time-dependent variation in expression among the four time points. Their expression indices and log_2 _expression ratios relative to time zero are tabulated in Additional File 1. The percent recovery of expression level was calculated as follows from the values of the log_2 _expression indices [log_2_(E)] for each gene:

$$\text{Percent recovery}=\left|\frac{\left(peak\log2(\text{E})-time\;10\log2(\text{E})\right)}{\left(peak\log2(\text{E})-time\;0\log2(\text{E})\right)}\right|\times 100$$

where "peak log_2_(E)" refers to the highest mean log_2_(E) of an up-regulated gene (at 1 min or at 5 min), or the lowest mean log_2_(E) of a down-regulated gene. For example, a gene whose expression increase peaks at 100-fold, then declines to 10-fold, would show 50% recovery with a remaining elevation of 10-fold; thus it would be "half recovered" in that the peak/recovered expression ratio equals the recovered/original expression ratio.

Genes that showed a significant increase or decrease in mean expression at 1 min and/or 5 min, and exhibited less than a 200% recovery (that is, over 100% past the baseline expression level), were defined as up-regulated or down-regulated, respectively (Additional File 1). Following the rapid acid shift, 630 genes (14% of the genome) were up-regulated and 586 genes (13%) were down-regulated. Their expression ratios relative to time zero, and their percent recovery, are tabulated. Of the genes defined as up-regulated or down-regulated, those whose expression recovered fully by 10 min (a percent recovery of between 100%–200%) were designated "transient." Thus defined, 52 genes (1.2% of the genome) showed transient up-regulation, and 45 genes (1%) showed transient down-regulation.

Genes that showed up-regulation or down-regulation by 5 min, and reached expression ratios of 2-fold or greater, are tabulated in Table 1 and Additional file 2, respectively. Many of these also show pH-dependent expression under steady-state conditions, in a study by Maurer *et al *in which the bacteria were cultured for several generations at pH 5.0, pH 7.0, and pH 8.7 [11]. The percentage of genes with elevated steady-state expression in acid culture [11] was 19%, for genes up-regulated at 1 min; 24%, at 5 min; and 34%, at 10 min (Table 1). For genes down-regulated after acid shift, the percentage down-regulated in steady-state acid culture [11] was 17% at 1 min, 17% at 5 min, and 27% at 10 min (Additional file 2). Thus, the longer a gene showed up-regulation (or down-regulation) in our study, the more likely it was to show elevated (or depressed) expression under steady-state growth at low pH.

**Table 1:** Genes upregulated at least 2-fold after acid shift.^a^

**1 min**	**Log2 ratios**	**Maurer et al 2005**^b^	**% Recovery**	**5 min**	**Log2 ratios**	**Maurer et al 2005**^b^	**% Recovery**	**10 min**	**Log2 ratios**	**Maurer et al 2005**^b^	**% Recovery**
*hisL*	2.3		66	*yagU*	4.3	+	10	*cadB*	4.7		0
*thrL*	1.7		77	*bdm*	4.0		20	*yagU*	3.8	+	10
*b2596*	1.6		90	*ymgB*	3.8		44	*glpD*	3.7		0
*raiA*	1.5		46	*glpD*	3.5		0	*glpK*	3.5		0
*YfiD*	1.5	+	52	*sra*	3.1	+	0	*hdeB*	3.5	+	0
*YifN*	1.4		75	*cadB*	3.1		0	*glpF*	3.3		0
*YifO*	1.4		87	*ymgA*	3.0		38	*hdeA*	3.3	+	0
*evgA*	1.3		45	*ymgC*	2.6		52	*sra*	3.3	+	0
*yhcN*	1.3	+	23	*yhcN*	2.6	+	23	*yahO*	3.2		0
*ymgB*	1.3		44	*yneI*	2.6		57	*bdm*	3.2		20
*uspD*	1.2		44	*gatB*	2.5	+	0	*cadA*	3.2	+	0
*ymgA*	1.2		38	*raiA*	2.5		46	*osmY*	3.1		0
*yagU*	1.1	+	10	*aceB*	2.4		33	*gatB*	3.1	+	0
*pmrD*	1.1		68	*b2596*	2.4		90	*lldR*	3.1		0
*ycgZ*	1.0		39	*uspD*	2.4	+	44	*gatC*	3.0	+	
				*gltA*	2.3	+	10	*yjbJ*	3.0		0
				*lldR*	2.3		0	*lldD*	2.9	+	0
				*gadE*	2.2		15	*yhjX*	2.8		0
				*lldD*	2.2	+	0	*gatA*	2.8	+	0
				*osmY*	2.2		0	*lldP*	2.8	+	0
				*glpF*	2.2		0	*rcsA*	2.7		0
				*ycgZ*	2.2		39	*yjdL*	2.6		0
				*osmB*	2.2		0	*gatZ*	2.6	+	0
				*rcsA*	2.1		0	*tnaC*	2.5		0
				*hdeB*	2.1	+	0	*glpQ*	2.5		0
				*gatA*	2.1	+	0	*clpB*	2.4	+	0
				*aceA*	2.1		8	*gcvT*	2.4		0
				*ygaC*	2.1		46	*ivy*	2.3		0
				*ivy*	2.0		0	*osmB*	2.3		0
				*yfiD*	2.0	+	52	*glpT*	2.3		0
				*yahO*	2.0		0	*dppF*	2.3		0
				*gatZ*	2.0		0	*msyB*	2.2		0
				*elbA*	2.0		59	*talA*	2.2		0
				*yjbJ*	1.9		0	*yiaG*	2.2		0
				*lldP*	1.9	+	0	*gcvH*	2.2		0
				*hisL*	1.9		66	*sucD*	2.2	+	0
				*amn*	1.9		27	*ymgB*	2.1		44
				*yeiG*	1.9	+	25	*gltA*	2.1	+	10
				*hdeA*	1.9	+	0	*gatY*	2.0		0
				*glpK*	1.9		0	*ydiZ*	2.0		0
				*gatC*	1.8			*yeaD*	2.0		
				*yhjX*	1.8		0	*yhcN*	2.0	+	23
				*crl*	1.8		64	*gadE*	1.9		15
				*hchA*	1.8		27	*ygaM*	1.9		0
				*gadW*	1.8		17	*aceA*	1.9		8
				*gltB*	1.7			*yhcO*	1.9		0
				*ilvC*	1.7		65	*dps*	1.9	+	0
				*arcB*	1.7		50	*ymgA*	1.9		38
				*evgA*	1.7		45	*ygdI*	1.8	+	0
				*uspA*	1.6		60	*gadB*	1.8		0
				*asd*	1.6		33	*bfr*	1.7		0
				*ghrB*	1.6		24	*gltB*	1.7		
				*osmC*	1.6		11	*ygiW*	1.7	+	0
				*cfa*	1.6	+	17	*sthA*	1.7	+	0
				*nlpA*	1.6	+	51	*aceB*	1.6		33
				*ycgK*	1.6		38	*yphA*	1.6		0
				*intA*	1.6		77	*elaB*	1.6		0
				*dps*	1.6	+	0	*yjiT*	1.6		0
				*rhsA*	1.6		30	*srlD*	1.6	+	0
				*fimB*	1.6		98	*spy*	1.6		0
				*yieF*	1.6	+	0	*aldA*	1.6	+	0
				*fpr*	1.6	+	47	*yieF*	1.6	+	0
				*yiiS*	1.6	+	23	*acs*	1.6		0
				*yieE*	1.5	+	33	*manY*	1.6		0
				*gatY*	1.5		0	*hdeD*	1.6		0
				*yhcO*	1.5		0	*dppA*	1.5		0
				*yggE*	1.5		0	*gadA*	1.5		0
				*insI*	1.5		47	*yeaG*	1.5		0
				*clpS*	1.5		62	*yggE*	1.5		0
				*glgC*	1.5		2	*mglB*	1.5		0
				*ydiZ*	1.5		0	*yncC*	1.5		0
				*dtd*	1.5		47	*b1005*	1.5		0
				*marA*	1.5	+	21	*gadW*	1.5		17
				*sthA*	1.4	+	0	*ykgA*	1.4	+	0
				*argE*	1.4		57	*osmC*	1.4		11
				*clpB*	1.4	+	0	*glgC*	1.4		2
				*serC*	1.4	+	57	*ytfK*	1.4		0
				*msyB*	1.4	+	0	*sdhB*	1.4	+	0
				*tas*	1.4	+	24	*dhaM*	1.4	+	0
				*ygdI*	1.4	+	0	*yeiG*	1.4	+	25
				*rsd*	1.4		67	*manZ*	1.4		0
				*aroG*	1.4		58	*ygaU*	1.4		0
				*bcsE*	1.4	+	37	*ykgE*	1.4		0
				*yciI*	1.4		74	*sdhC*	1.4	+	0
				*gcvR*	1.4		81	*dadA*	1.4		
				*nrdH*	1.3		78	*amn*	1.4		27
				*bcsF*	1.3		31	*yodC*	1.4	+	0
				*ygdR*	1.3		22	*manX*	1.4		0
				*acnB*	1.3	+	15	*udp*	1.4	+	0
				*appY*	1.3		36	*fumA*	1.4	+	0
				*ypeC*	1.3		36	*glgS*	1.4		0
				*yrdA*	1.3		29	*ycgZ*	1.3		39
				*hdfR*	1.3		73	*cfa*	1.3	+	17
				*yjdJ*	1.3		4	*ybjP*	1.3		0
				*thrL*	1.3		77	*raiA*	1.3		46
				*umC*	1.3		5	*oppB*	1.3		
				*yncC*	1.3		0	*yehE*	1.3		0
				*yjiT*	1.3		0	*uspD*	1.3	+	44
				*gcvT*	1.3		0	*mqo*	1.3	+	0
				*ugpB*	1.3		25	*oppA*	1.3		
				*ygbE*	1.3	+	1	*sdhA*	1.3	+	0
				*hdfR*	1.3		65	*hdhA*	1.3		0
				*hdhA*	1.3		0	*hchA*	1.3		27
				*yjgK*	1.3	+	41	*yhhA*	1.3		0
				*cadA*	1.2	+		*dppD*	1.3		0
				*rpoS*	1.2			*poxB*	1.3		0
				*oppB*	1.2			*srlE*	1.3	+	0
				*dadA*	1.2			*yjdI*	1.2		0
				*oppA*	1.2			*ygbE*	1.2	+	1
				*ybjP*	1.2		0	*ghrB*	1.2		24
				*sbmC*	1.2		47	*yjdJ*	1.2		4
				*malP*	1.2		55	*ymgC*	1.2		52
				*msrA*	1.2		50	*mglA*	1.2		0
				*bcp*	1.2		72	*fumC*	1.2		5
				*arcA*	1.2		47	*wrbA*	1.2		0
				*mqo*	1.2	+	0	*ykgF*	1.2		0
				*ycbB*	1.2		42	*yqjD*	1.2		0
				*yjdL*	1.2		0	*cdd*	1.2	+	0
				*yhhA*	1.2		0	*yiiS*	1.2	+	23
				*malQ*	1.2		58	*yqjC*	1.2		0
				*nadE*	1.2	+	39	*groL*	1.2	+	0
				*aldA*	1.2	+	0	*ygaF*	1.2		0
				*cyaA*	1.2	+	73	*otsB*	1.2		0
				*dkgA*	1.2		9	*marA*	1.1	+	21
				*marR*	1.2	+	28	*htpG*	1.1	+	0
				*glgS*	1.2		0	*acnB*	1.1	+	15
				*yeaR*	1.2		11	*srlA*	1.1	+	0
				*gdhA*	1.2		54	*ybeL*	1.1		0
				*ykgA*	1.2	+	0	*ygaC*	1.1		46
				*yfaO*	1.2		34	*asd*	1.1		33
				*soxS*	1.2		85	*dkgA*	1.1		9
				*ygiN*	1.2		63	*rhsA*	1.1		30
				*fucK_*	1.2		65	*ydiH*	1.1	+	0
				*yodA*	1.2		79	*dhaK*	1.1	+	0
				*sucD*	1.2	+	0	*yneI*	1.1		57
				*argT*	1.2		58	*sdhD*	1.1	+	0
				*ygaM*	1.2		0	*dhaL*	1.1		0
				*rhsD*	1.2		108	*yhdN*	1.1		6
				*fxsA*	1.1			*yeaQ*	1.1	+	0
				*yqhD*	1.1			*uspB*	1.1		0
				*yihY*	1.1			*yeaR*	1.1		11
				*yhdN*	1.1		6	*gltD*	1.1	+	0
				*gloB*	1.1		46	*tas*	1.0	+	24
				*hdeD*	1.1		0	*ygdR*	1.0		22
				*sdhA*	1.1	+	0	*sodC*	1.0		0
				*yiaG*	1.1		0	*yieE*	1.0	+	33
				*zntR*	1.1		23	*galP*	1.0		0
				*yjeI*	1.1		84	*fliY*	1.0	+	0
				*ldhA*	1.1		37	*nemA*	1.0		
				*yqhD*	1.1		18				
				*srlD*	1.1	+	0				
				*yihY*	1.1		18				
				*yeaC*	1.1	+	19				
				*gcvH*	1.1		0				
				*talA*	1.1		0				
				*yphA*	1.1		0				
				*ybaQ*	1.1		96				
				*nrdI*	1.1		80				
				*ygiW*	1.1	+	0				
				*yfjU*	1.1		11				
				*elaB*	1.1		0				
				*yjdC*	1.1		16				
				*yfbU*	1.1		46				
				*bcsG*	1.1		34				
				*sdhC*	1.1	+	0				
				*yohN*	1.1	+	11				
				*bolA*	1.1		65				
				*yjdI*	1.1		0				
				*sdhB*	1.1	+	0				
				*ydiV*	1.1		62				
				*mcrB*	1.1		15				
				*yggG*	1.1		18				
				*dppA*	1.1		0				
				*mcrC*	1.1		12				
				*nemA*	1.0						
				*yeaD*	1.0						
				*ygaU*	1.0		0				
				*yjbQ*	1.0	+	55				
				*yfbQ*	1.0	+	52				
				*yihD*	1.0		65				
				*ybeL*	1.0		0				
				*phoP*	1.0		44				
				*nlpD*	1.0		20				
				*gadB*	1.0		0				
				*uspB*	1.0		0				
				*rpoN*	1.0		56				
				*yfjH*	1.0		38				
				*yaiB*	1.0		8				
				*spy*	1.0		0				
				*betI*	1.0		138				
				*lrp*	1.0		44				
				*ybhGt*	1.0		74				
				*gadX*	1.0		23				
				*ygiT*	1.0		101				
				*kch*	1.0		47				
				*ygdH*	1.0		37				

^a ^Values shown represent the log_2 _ratio of expression indices compared to those at time zero. Expression ratios of 2-fold or greater are shown. Percent recovery is defined under Results; a blank cell indicates recovery could not be defined.

^b ^Ref [11]

Genes whose up-regulation (or down-regulation) was transient would not be expected to show steady-state expression ratios. In fact, of the 52 genes showing transient up-regulation to levels 2-fold or greater, none are reported in Ref. [11]; and of the 45 genes showing transient down-regulation, only one is reported. Thus, our study revealed transiently acid-responsive genes that were not observed under steady-state growth conditions.

### Up-regulated genes following an acid shift

#### *yagU *and other acid stress genes of unknown function

The gene encoding inner-membrane protein YagU [24] contributes to acid resistance [12] and is one of the genes most strongly up-regulated (16-fold) at low pH [11,12]. Following acid shift, *yagU *was one of the top genes up-regulated at 1 min, with peak expression at 5 min and a 10% recovery at 10 min (Table 1).

**Figure 2:**
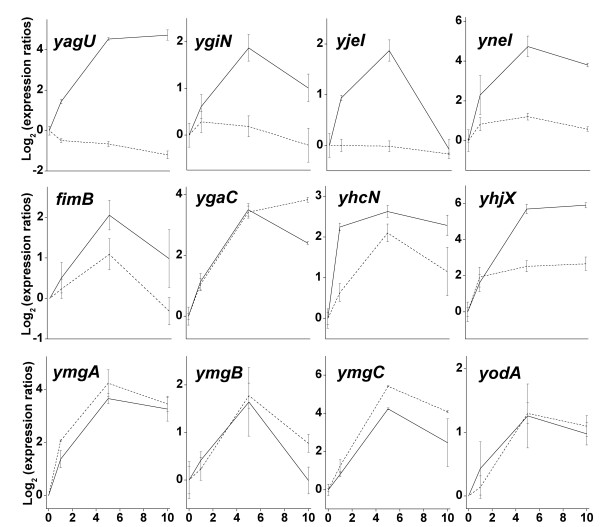
**Real-Time PCR expression ratios of selected genes**. Bacteria were cultured and real-time PCR was conducted as described in Materials and Methods. For each experimental condition, the 1, 5, and 10 min expression values were normalized to time 0. For each gene, log_2 _(expression ratios) are shown for amplified message after the rapid addition of either HCl (15 mM, solid line) or sodium benzoate (30 mM, dotted line).

The up-regulation of *yagU *was confirmed by real-time PCR (Fig. 2). The time course of *yagU *expression relative to time 0 was similar to that of the microarray data (Table 1). In order to determine whether up-regulation was related to external pH or cytoplasmic pH, a second time course was conducted using the permeant weak acid benzoate. Addition of benzoate to cultures suspended at pH_ext _7.0 acidifies the cytoplasmic pH while the external pH remains constant. The *yagU *gene showed no significant up-regulation following benzoate addition. Therefore, *yagU *response is more consistent with direct sensing of external pH, not cytoplasmic pH.

Three other genes observed by real-time PCR (*ygiN*, *yjeI*, and *yneI*) showed approximately 16-fold increase in expression after rapid acid shift, but no effect of benzoate addition (Fig. 2). Expression of *yjeI *recovered after acid shift, but the benzoate experiment ruled out a direct response to cytoplasmic pH. Eight other genes tested (*fimB*, *ygaC*, *yhcN*, *yhjX*, *ymgA*,*ymgB*,*ymgC*, and *yodA*) increased expression more than 4-fold with full or partial recovery, and were up-regulated after benzoate addition. This pattern is consistent with response to intracellular pH change. The gene *fimB *encodes the flagellar phase switching mediator FimB [25]; the others have unknown functions. None of these genes were previously reported to be up-regulated by acid.

#### Acid-inducible amino-acid decarboxylases

The most well-documented class of response to external acid is the degradative amino-acid decarboxylases, which convert specific amino acids to amines while exporting a proton [14,26]. All of these were up-regulated within 5 min following the acid shift, with little or no recovery. These include the lysine decarboxylase and transporter *cadBA *[27] and the arginine decarboxylase *adiY *[28] (see Table 1 and Additional File 1) as well as the glutamate decarboxylase and transporter (*gadA *and *gadBC*) which are part of the *gad *acid response regulon [9,29]. Other members of the *gad *regulon up-regulated after the acid shift included *gadE*, *gadWX*, and *evgAS*.

#### Metabolism and transport

A number of metabolic enzymes and transporters were up-regulated following acid shift. Several of these are associated with biofilm formation, such as isocitrate lyase (*aceA*) and malate synthase (*aceB*) [30]. The proteins of galactitol transport were upregulated strongly (*gatA*, *B*, *Y*, *Z*). The *gat *genes are involved in biofilm formation [30] and they show elevated expression in steady-state acid, which appears to favor substrates that produce fewer acidic fermentation products [11,12]. Biofilm development genes *ymgABC *were also up-regulated after acid shift, as was the biofilm-dependent modulation protein *bdm *[31]. The expression levels of *ymgABC *began recovery by 10 min. These genes have not been reported previously to show elevated expression under steady-state acid conditions.

Other metabolic genes known for acid-enhanced expression [11,12] were found to be up-regulated without recovery following acid shift. These include *sdhABCD*, encoding succinate dehydrogenase, [32]; the *dppA, B, C, D, F *dipeptide transport system [33]; and glycerol transport components *glpD*, *F*, *Q*, *T*, of which *glpD *encodes a multidrug-resistance component associated with persister cell formation [34].

#### Osmotic shock response genes

Several genes up-regulated after acid shift are associated with osmotic shock, in particular the Rcs regulon [31,35]. In the Rcs regulon, the transcriptional regulators RcsA and RcsB control expression of genes involved in colanic capsule biosynthesis. The Rcs regulon members up-regulated after acid shift included *rcsABCD*, *bdm*, and *osmB *[36]. Of these, only *rcsA *shows elevated expression in steady-state acid [11]. An osmotic shock gene outside the Rcs regulon, *osmY *[37], also was up-regulated after acid shift.

#### The Fur regulon

The ferric uptake regulator (Fur) governs the expression of various acid tolerance genes [38]. Of the genes that were strongly-up-regulated after rapid acidification, eight are genes previously shown to be controlled by the Fur regulon [39,32,42], Genes whose expression peaked at 5 min encode the cadmium induced metal binding protein (*yodA*) [39], *ygaC *[40], and the oxidative stress genes *nrdH *and *nrdI *[42]. The percent recovery of these genes ranged between 46 to 80% (Table 1). Fur also regulates the succinate dehydrogenase operon *sdhABCD*, which is up-regulated by acid under steady-state conditions [11] and was up-regulated after rapid acidification (Table 1).

Many genes of unknown or putative function exhibited expression profiles that paralleled the shift and recovery of cytoplasmic pH. Such genes include *ygiN*, whose gene product has recently been isolated and characterized as a quinol monooxygenase [43]. The gene *ygiN *exhibited peak up-regulation at 5 min and made a 63% recovery by 10 min (Table 1). Genes encoding the putative NAD^+^- dependent succinate semialdehyde dehydrogenase YneI [44] and the putative periplasmic protein YhcN, exhibited peak expression at 5 min and recovered by 57% and 23% respectively (Table 1).

### Down-regulated genes following an acid shift

The most strongly down-regulated genes following acid shift are shown in Additional file 2. Many of these show steady-state elevated expression at high pH.

Genes encoding the high-affinity cytochrome oxidoreductase *d *(*cyd *operon) are favored under low-oxygen conditions as well as at high pH [11]. The genes *cydA, B*, *C *were down-regulated following acid shift. Similarly, the periplasmic extracytoplasmic response regulator CpxP [45,46] is strongly up-regulated in base [11]. The *cpxP *gene was down-regulated immediately after acid shift, with little recovery (Additional file 2). The gene encoding the base-inducible putative membrane-bound redox modulator Alx [11,47,48] was also down-regulated, as were the genes encoding serine deaminase and transport, *sdaABC *(Additional Files 1 and 2). Amino-acid deaminases appear to be induced as a response to reverse pH increase [11,18,49].

Many genes involved in polyamine metabolism and transport show increased expression at high pH [11,50]. Several of these were down-regulated following acid shift, including the spermidine metabolism genes *speA*, *B*, *D*, *E *[51] and polyamine transport genes *potA, B, C, D *[52].

### Delayed response to acid shift

Some genes showed little or no response to acid shift until the 10-min time point (included in Additional File 1). Most striking was the delayed up-regulation of the *nuo *operon encoding NADH dehydrogenase I [53,54]. Thirteen genes of the *nuo *operon were all up-regulated about 50% at 10 min, after little or no significant differences at 1 min or 5 min. The NADH dehydrogenase I is known to show elevated expression in steady-state acid [11] and under anoxic conditions; its expression declines with aeration [55]. Our new data showed that *nuo *expression does not increase immediately with acidity, but as a delayed consequence of the acid shift. The previously reported rise in NADH dehydrogenase could be a consequence of deenergization following acid stress. Another complex showing delayed up-regulation by acid was the HslVU protease [56]. The *hsl *genes are known to be up-regulated by acid under steady-state conditions [11]. Our new data is more consistent with a secondary response to acidification, such as a need to degrade acid-denatured proteins.

## Conclusion

When cultures of *E. coli *K-12 W3110 were subjected to a rapid decrease in external pH, from pH 7.6 to pH 5.5, the cytoplasmic pH decreased but began to recover within less than a minute after the acid shift. Up-regulated genes included amino-acid decarboxylases (*cadA, adiY*, *gadA*), succinate dehydrogenase (*sdhABCD*), biofilm-associated genes (*bdm*, *gatAB*, and *ymgABC*), and components of the Gad, Fur and Rcs regulons. Many of the acid-up-regulated genes showed sustained elevation, such as *cadA *and *sdhCD*. On the other hand, the rapid acid exposure experiment revealed genes whose response to acid shift is transient, such as the biofilm development genes *ymgABC *and the flagellar phase variation modulator *fimB*. These genes were up-regulated after benzoate addition, a condition in which cytoplasmic pH is depressed permanently without change in external pH. Thus, these genes are candidates for direct response to cytoplasmic acid stress. The acid response of other genes, such as *nuo *and *hsl*, was shown to be delayed. Their delayed response suggests a secondary effect of growth in acid. Overall, our study of gene expression following rapid acid shift or rapid benzoate addition revealed several classes of acid stress response: genes showing sustained up-regulation (or down-regulation) following acid shift; genes showing transient up-regulation after acid shift, as well as up-regulation following permeant-acid treatment of the cytoplasm; and genes showing delayed up-regulation after acid shift.

## Methods

### Growth conditions

#### Rapid external acid stress

*Escherichia coli *K-12 strain W3110 [57] was cultured overnight in buffered LBK medium [11]. Overnight cultures were diluted 550-fold into 55 mLs of buffered LBK medium and aerated at 225 rpm in an orbital water bath. The medium was buffered with 20 mM homopiperazine-N, N'-bis-2-(ethane-sulfonic acid) (HOMOPIPES) (pK_a _4.55), and was adjusted using KOH to pH 7.6. Cultures were grown at 37°C to an OD_600 _of 0.2 (approximately five generations). At OD_600 _= 0.2, the pH of the medium was lowered to pH 5.5 with the addition of 1 M HCl (840 μL). One 10-mL sample was taken from each of the five biological replicates immediately before the addition of HCl (0 min). After HCl addition, three 10-mL samples were taken from each of the five biological replicates, corresponding to 1, 5, and 10 min. The pH of the cultures was tested at the end of the acid shift to ensure they were maintained within 0.2 pH units of pH 5.5.

#### Permeant acid stress

The permeant-acid time course was performed in order to generate a permanent intracellular acidification, thus revealing genes responsive to cytoplasmic pH stress. The growth conditions were the same as those used for HCl acidification, with the exception of the pH and buffering capacity of the medium. The medium was adjusted to pH 7.0 with KOH solution and was buffered with 50 mM HOMOPIPES (pK_a _4.55). The external pH was set at 7.0 in order to ensure that the addition of weak acid led to a permanent decrease in cytoplasmic pH (from a cytoplasmic pH of approximately 7.6). Sodium benzoate (pH 7.2) was added once cultures reached an OD_600 _of 0.2, for a final concentration of 30 mM. As described above, samples were taken at time 0, 1, 5, and 10 min. The pH of the media remained at pH 7.0 ± 0.2. RNA was extracted to be analyzed by real-time PCR.

### Cytoplasmic pH Measurement

Cytoplasmic pH was measured based on YFP fluorescence as reported [16]. Measurements were made on strain JLS0617, which consists of strain W3110 transformed with pSL38-YFP containing *tetR*-YFP [58]. Strain JLS0617 was cultured overnight in buffered M63 salts medium [20 mM homopiperazine-*N*,*N*-bis-2-(ethanesulfonic acid) (HOMOPIPES), pH 7.6] containing 1.5% casein hydrolysate, 0.8% glycerol, and 100 μg/ml ampicillin. The overnight culture was diluted 25-fold in the same medium and cultured to late log phase (OD_600 _= 0.8 to 0.9) at 37°C with aeration. The cultures were suspended to an OD_600 _of 0.4 in M63 medium containing 0.2% casein hydrolysate, 0.8% glycerol, and 20 mM HOMOPIPES (pH 7.5). The suspension media used for the permeant acid experiment contained 50 mM HOMOPIPES, in order to ensure that the external pH was maintained at pH 7.0.

Excitation spectra were recorded using a Fluoromax-3 spectrofluorimeter (Horiba Jobin Yvon). A cell suspension (3 ml) was placed into a Starna Spectrosil quartz cuvette with a path length of 10 mm. The temperature of the chamber was adjusted to 30°C, and aeration was provided by stirring. Spectra were recorded at 30°C because the YFP probe is temperature sensitive and exhibits the highest fluorescence signal at this temperature. YFP excitation was measured from 490 to 520 nm (slit width, 2 nm), using an emission wavelength of 550 nm (slit width, 20 nm). Continuous excitation spectra were obtained every 4 s for 1 min before aliquot addition (time zero) and then for 10 min after addition. In order to control for effects on fluorescence unrelated to pH change, KCl (8.5 mM, pH 7.6) was added to cell suspensions at time 0. For each condition, spectra were recorded for three biological replicates.

Data were fitted to a standard curve correlating cytoplasmic pH with fluorescence intensity. A linear interpolation was conducted between pH 7.6 and pH 5.5 signal intensities. The fluorescence corresponding to pH 5.5 was determined by adding an additional 20 mM benzoate to each test sample at the end of the time course (thus collapsing the cytoplasmic pH to pH 5.5.)

The pH curve was modified for experiments in which a permeant acid was added to cultures in medium at external pH 7.0. In this case, linear interpolation was conducted between pH 7.6 and pH 7.0. The fluorescence corresponding to pH 7.0 was determined by adding an additional 20 mM benzoate (50 mM benzoate was the final concentration) to each test sample at the end of the time course.

### RNA isolation

Samples were transferred from growth culture to vials containing 1 mL ice-cold 10% phenol-ethanol stop solution [59] in order to stabilize the bacterial RNA. RNA was isolated as described previously [11,12] using the RNeasy Kit with on-column DNA digestion (Qiagen). The quality of the isolated RNA was examined using the Agilent Bioanalyzer 2100.

### cDNA preparation and array hybridization

Standard methods were used for cDNA synthesis, fragmentation, and end-terminus biotin labeling [11,12]. Labeled cDNA samples were hybridized to Affymetrix GeneChip *E. coli *Antisense Genome Arrays. Hybridized arrays were stained with streptavidin-phycoerythrin using the Affymetrix Fluidic Station 450. After staining, arrays were scanned with a GC3000 scanner.

### Analysis of Gene Expression

Log_2 _transformed model-based expression values were calculated from the probe level data using D-Chip software [19]. In order to determine what genes show significant time dependent variation in expression following the acid shift over 1, 5, and 10 min, a mixed effects linear model for longitudinal data was performed using Statistical Analysis System (SAS) [20-22]. A p-value < 0.001 was considered a significant time dependence, which corresponds to a false positive rate of 1 in 1000 genes.

Log_2 _ratios for expression indices at times 1, 5, and 10 minutes post HCl addition were determined for each gene by subtracting the time 0 log_2_(E) from the time 1, 5, and 10 minute log_2_(E) respectively.

### Real-Time Polymerase Chain Reaction (PCR)

Expression of mRNA for individual genes was quantified by real-time PCR using an ABI Prism 7500 DNA analyzer (Applied Biosystems) as performed previously [12]. Primer Express Software v2.0 (Applied Biosystems) was used for primer design. The primers chosen had minimal GC content and amplified 50–70 bp segments of the target genes (Table 2). The SYBR Green PCR One-Step RT-PCR protocol (Applied Biosystems) was used, in which cDNA reverse transcription and PCR amplification occur in the same well. Nucleic acid concentrations were: 0.1 nM forward primer, 0.1 nM reverse primer, and 50 ng target RNA. PCR cycling conditions were: reverse transcription at 48°C for 30 min, 95°C for 10 min, 40 cycles of denaturation at 92°C for 15s, and extension at 60°C for 1 min. For detection of primer dimerization or other artifacts of amplification, a dissociation curve was run immediately after completion of the real-time PCR. Individual gene expression profiles were normalized based on measurement of the original RNA sample amplified. All expression levels are presented relative to the expression at time 0.

**Table 2:** Primers for real-time PCR

**Gene Name**	**Forward Primer Sequence**	**Reverse Primer Sequence**
*fimB*	5'-TCGTACCCGCATGCTGAGA-3'	5'-AAAGCGGATTCCCCTTACGT-3'
*yagU*	5'-TCCATTGCCGCCACGTA-3'	5'-GGGCCACACGCTGCAT-3'
*ygaC*	5'-GCGAGCGACACGAGCATT-3'	5'-AAATAACGTTCAAGCGCAACAC-3'
*ygiN*	5'-TCTTGAAGCGCATCTGCAAA-3'	5'-CCTTTTACGGCTTCGCTATACG-3'
*yhcN*	5'-GCGTCTTCGCCAATGGAT-3'	5'-GCCGTTGCGCCTTTCTC-3'
*yhjX*	5'-TCCATCGCCAACCTTTCAG-3'	5'-GGATACGGGCGATTTTGTCA-3'
*YjeI*	5'-CAGGCGGCTGCAATGG-3'	5'-CTGCGACGGGCTACTGATG-3'
*ymgA*	5'-CCGACGCGATTTAATTGACA-3'	5'-TTTGGCCCTCCTGAATCACT-3'
*ymgB*	5'-AGTTACTTTCGCAGTTCGGGTAA-3'	5'-GGTGACAGCCTGCCCTAACA-3'
*ymgC*	5'-CTCGAGAGGGAGGTGTTCATG-3'	5'-GCGTAAAGCCTGATCAATAATATGAC-3'
*yneI*	5'-GCGAAATGGGCAAACCAA-3'	5'-GCCGATTTCGCCACTTCA-3'
*yodA*	5'-GGTGTTTTTGATGATGCCAATG-3'	5'-GCCAGACTCCATCCCAGTCA-3'

## Authors' contributions

GK and JCW designed and conducted the experiments and authored the first draft manuscript. DMF contributed real-time PCR assays. BDJ devised and conducted the statistical analysis. SSB directed the array hybridization. JLS conceived the study, coordinated the project overall, and revised the final manuscript. All authors have read and approved the final manuscript.

## Supplementary Material

Additional file 1 Expression indices, expression ratios and percent recovery of genes that show time-dependent expression in response to a rapid change in external pH. Click here for file

Additional file 2 Table showing Genes downregulated at least 2-fold after acid shift. Click here for file
