# Supplementary material for: Rapid acid treatment of Escherichia coli: transcriptomic response and recovery
Source: BMC Microbiol. 2008 Feb 26;8:37. doi: 10.1186/1471-2180-8-37 (PMC2270276; doi:10.1186/1471-2180-8-37)
Supplement: Additional file 2 — Table showing Genes downregulated at least 2-fold after acid shift. [file 1471-2180-8-37-S2.doc]

| **Table 2** | | | | | | | | | | | |
| --- | --- | --- | --- | --- | --- | --- | --- | --- | --- | --- | --- |
| **Genes downregulated at least 2-fold after acid shift.^a^** | | | | | | | | | | | |
| **1 min** | **Log2 ratios** | **Maurer et al 2005^b^** | **% Recovery** | **5 min** | **Log2 ratios** | **Maurer et al 2005^b^** | **% Recovery** | **10 min** | **Log2 ratios** | **Maurer et al 2005^b^** | **% Recovery** |
| *cpxP* | -1.4 | + | 34 | *sdaC* | -3.4 | + | 27 | *ompF* | -2.6 |  |  |
| *b3914* | -1.3 |  | 12 | *yeeF* | -2.7 |  | 21 | *acfD* | -2.6 | + | 0 |
| *glnW* | -1.1 |  | 88 | *fadL* | -2.6 | + | 5 | *sdaC* | -2.5 | + | 27 |
| *rsxB* | -1.1 |  | 58 | *acfD* | -2.5 | + | 0 | *fadL* | -2.5 | + | 5 |
| *argX* | -1.1 |  | 68 | *speE* | -2.4 |  | 21 | *yeeF* | -2.1 |  | 21 |
| *ybeB* | -1.0 |  | 24 | *potB* | -2.3 |  | 36 | *speD* | -2.0 | + | 6 |
|  |  |  |  | *rpsF* | -2.3 |  | 77 | *yghJ* | -2.0 | + | 0 |
|  |  |  |  | *greA* | -2.2 |  | 38 | *dusB* | -1.9 |  |  |
|  |  |  |  | *potA* | -2.2 |  | 34 | *speE* | -1.9 |  | 21 |
|  |  |  |  | *hepA* | -2.2 |  | 39 | *fis* | -1.9 |  | 0 |
|  |  |  |  | *speD* | -2.1 | + | 6 | *yccA* | -1.8 | + | 0 |
|  |  |  |  | *rnpA* | -2.0 |  | 59 | *borD* | -1.6 | + | 0 |
|  |  |  |  | *yegQ* | -2.0 |  | 25 | *mutL* | -1.6 | + | 16 |
|  |  |  |  | *dusB* | -1.9 |  |  | *lysP* | -1.6 |  | 0 |
|  |  |  |  | *rluA* | -1.9 |  | 28 | *ydfO* | -1.6 |  | 0 |
|  |  |  |  | *pyrD* | -1.9 |  | 63 | *sdaB* | -1.5 | + | 9 |
|  |  |  |  | *b3913* | -1.9 | + | 34 | *potB* | -1.5 |  | 36 |
|  |  |  |  | *pitA* | -1.9 |  | 31 | *yegQ* | -1.5 |  | 25 |
|  |  |  |  | *mutL* | -1.9 | + | 16 | *potA* | -1.4 |  | 34 |
|  |  |  |  | *rpsR* | -1.9 |  | 79 | *deaD* | -1.4 |  |  |
|  |  |  |  | *yghJ* | -1.9 | + | 0 | *cpxP* | -1.4 | + | 12 |
|  |  |  |  | *rplS* | -1.8 |  |  | *rluA* | -1.4 |  | 28 |
|  |  |  |  | *mltD* | -1.8 | + | 24 | *mltD* | -1.4 | + | 24 |
|  |  |  |  | *rsxB* | -1.8 |  | 58 | *bioB* | -1.4 | + | 0 |
|  |  |  |  | *rbfA* | -1.8 |  | 54 | *greA* | -1.4 |  | 38 |
|  |  |  |  | *ydiY* | -1.8 |  | 58 | *nupG* | -1.3 |  | 18 |
|  |  |  |  | *yqgB* | -1.8 | + | 59 | *pitA* | -1.3 |  | 31 |
|  |  |  |  | *pheS* | -1.7 |  | 36 | *hepA* | -1.3 |  | 39 |
|  |  |  |  | *prfC* | -1.7 |  | 55 | *nmpC* | -1.3 |  |  |
|  |  |  |  | *yidC* | -1.7 |  | 41 | *chaA* | -1.3 |  | 0 |
|  |  |  |  | *priB* | -1.7 |  | 77 | *yghG* | -1.3 |  | 17 |
|  |  |  |  | *queA* | -1.7 |  | 45 | *apt* | -1.3 |  | 22 |
|  |  |  |  | *rplK* | -1.7 |  | 85 | *b3913* | -1.3 | + | 34 |
|  |  |  |  | *rpsO* | -1.7 |  | 53 | *pstS* | -1.3 |  |  |
|  |  |  |  | *alaU* | -1.7 |  | 71 | *pstB* | -1.2 |  | 0 |
|  |  |  |  | *fis* | -1.7 |  | 0 | *potC* | -1.2 |  | 21 |
|  |  |  |  | *sdaB* | -1.6 | + | 9 | *rpsT* | -1.1 |  | 24 |
|  |  |  |  | *yhbC* | -1.6 |  | 59 | *alx* | -1.1 |  | 5 |
|  |  |  |  | *nupG* | -1.6 |  | 18 | *pheS* | -1.1 |  | 36 |
|  |  |  |  | *rpsP* | -1.6 |  | 70 | *pppA* | -1.1 |  | 25 |
|  |  |  |  | *apt* | -1.6 |  | 22 | *ndh* | -1.1 |  | 0 |
|  |  |  |  | *cpxP* | -1.6 | + | 12 | *nlpI* | -1.1 |  | 30 |
|  |  |  |  | *yccA* | -1.6 | + | 0 | *typA* | -1.1 |  | 28 |
|  |  |  |  | *lysP* | -1.6 |  | 0 | *ttcA* | -1.1 |  | 24 |
|  |  |  |  | *nlpI* | -1.6 |  | 30 | *bioC* | -1.0 |  | 0 |
|  |  |  |  | *deaD* | -1.5 |  |  | *nrdA* | -1.0 |  | 0 |
|  |  |  |  | *borD* | -1.5 | + | 0 | *yfhL* | -1.0 |  | 10 |
|  |  |  |  | *yghG* | -1.5 | + | 17 | *yidC* | -1.0 |  | 41 |
|  |  |  |  | *rpsT* | -1.5 |  | 24 | rplT | -1.0 |  |  |
|  |  |  |  | *yjjJ* | -1.5 |  | 59 |  |  |  |  |
|  |  |  |  | *typA* | -1.5 |  | 28 |  |  |  |  |
|  |  |  |  | *lepA* | -1.5 |  | 38 |  |  |  |  |
|  |  |  |  | *potC* | -1.5 |  | 21 |  |  |  |  |
|  |  |  |  | *pppA* | -1.5 | + | 25 |  |  |  |  |
|  |  |  |  | *fadE* | -1.5 |  | 49 |  |  |  |  |
|  |  |  |  | *rpsI* | -1.4 |  |  |  |  |  |  |
|  |  |  |  | *ydfO* | -1.4 |  | 0 |  |  |  |  |
|  |  |  |  | *rpsH* | -1.4 |  | 80 |  |  |  |  |
|  |  |  |  | *infA* | -1.4 |  | 41 |  |  |  |  |
|  |  |  |  | *alaT* | -1.4 |  | 70 |  |  |  |  |
|  |  |  |  | *yfgB* | -1.4 |  | 52 |  |  |  |  |
|  |  |  |  | *ttcA* | -1.4 |  | 24 |  |  |  |  |
|  |  |  |  | *rfbC* | -1.4 | + | 49 |  |  |  |  |
|  |  |  |  | *rpmH* | -1.4 |  | 50 |  |  |  |  |
|  |  |  |  | *intR* | -1.4 |  | 38 |  |  |  |  |
|  |  |  |  | *yadB* | -1.4 |  | 59 |  |  |  |  |
|  |  |  |  | *yghF* | -1.4 |  | 33 |  |  |  |  |
|  |  |  |  | *tsgA* | -1.3 |  | 85 |  |  |  |  |
|  |  |  |  | *nmpC* | -1.3 |  |  |  |  |  |  |
|  |  |  |  | *prs* | -1.3 | + | 71 |  |  |  |  |
|  |  |  |  | *ytfF* | -1.3 |  | 26 |  |  |  |  |
|  |  |  |  | *ygiQ* | -1.3 |  | 50 |  |  |  |  |
|  |  |  |  | *ybcV* | -1.3 |  | 34 |  |  |  |  |
|  |  |  |  | *rplF* | -1.3 |  | 79 |  |  |  |  |
|  |  |  |  | *rpsN* | -1.3 |  | 82 |  |  |  |  |
|  |  |  |  | *rpmD* | -1.3 |  | 71 |  |  |  |  |
|  |  |  |  | *pssA* | -1.3 |  | 44 |  |  |  |  |
|  |  |  |  | *nusA* | -1.3 |  | 59 |  |  |  |  |
|  |  |  |  | *ycbY* | -1.3 |  | 43 |  |  |  |  |
|  |  |  |  | *rpsQ* | -1.3 |  | 28 |  |  |  |  |
|  |  |  |  | *rnb* | -1.3 |  | 30 |  |  |  |  |
|  |  |  |  | *plsX* | -1.3 |  | 30 |  |  |  |  |
|  |  |  |  | *gsk* | -1.3 |  | 35 |  |  |  |  |
|  |  |  |  | *srmB* | -1.3 |  | 41 |  |  |  |  |
|  |  |  |  | *nupC* | -1.2 |  | 96 |  |  |  |  |
|  |  |  |  | *cspA* | -1.2 |  | 27 |  |  |  |  |
|  |  |  |  | *ispU* | -1.2 |  | 54 |  |  |  |  |
|  |  |  |  | *yeaZ* | -1.2 |  | 35 |  |  |  |  |
|  |  |  |  | *secE* | -1.2 |  | 51 |  |  |  |  |
|  |  |  |  | *chaA* | -1.2 |  | 0 |  |  |  |  |
|  |  |  |  | *cmk* | -1.2 |  | 35 |  |  |  |  |
|  |  |  |  | *nth* | -1.2 |  | 37 |  |  |  |  |
|  |  |  |  | *aroM* | -1.2 |  | 41 |  |  |  |  |
|  |  |  |  | *der* | -1.2 |  | 40 |  |  |  |  |
|  |  |  |  | *rsxD* | -1.2 |  | 44 |  |  |  |  |
|  |  |  |  | *gidB* | -1.2 |  | 39 |  |  |  |  |
|  |  |  |  | *suhB* | -1.2 |  | 44 |  |  |  |  |
|  |  |  |  | *alx* | -1.2 | + | 5 |  |  |  |  |
|  |  |  |  | *nusG* | -1.2 |  | 57 |  |  |  |  |
|  |  |  |  | *pnp* | -1.2 |  | 47 |  |  |  |  |
|  |  |  |  | *rsxG* | -1.2 |  | 44 |  |  |  |  |
|  |  |  |  | *rsxC* | -1.2 |  | 39 |  |  |  |  |
|  |  |  |  | *ybeB* | -1.2 |  | 24 |  |  |  |  |
|  |  |  |  | *fhuA* | -1.2 |  | 24 |  |  |  |  |
|  |  |  |  | *ycjZ* | -1.2 |  | 36 |  |  |  |  |
|  |  |  |  | *tgt* | -1.2 | + | 16 |  |  |  |  |
|  |  |  |  | *ompF* | -1.1 |  |  |  |  |  |  |
|  |  |  |  | *rplT* | -1.1 |  |  |  |  |  |  |
|  |  |  |  | *pstS* | -1.1 |  |  |  |  |  |  |
|  |  |  |  | *rplX* | -1.1 |  | 82 |  |  |  |  |
|  |  |  |  | *dnaE* | -1.1 |  | 24 |  |  |  |  |
|  |  |  |  | *ispE* | -1.1 | + | 80 |  |  |  |  |
|  |  |  |  | *dusC* | -1.1 |  | 36 |  |  |  |  |
|  |  |  |  | *rfbX* | -1.1 |  | 50 |  |  |  |  |
|  |  |  |  | *yccS* | -1.1 |  | 21 |  |  |  |  |
|  |  |  |  | *yccF* | -1.1 |  | 27 |  |  |  |  |
|  |  |  |  | *bioB* | -1.1 | + | 0 |  |  |  |  |
|  |  |  |  | *treC* | -1.1 | + | 128 |  |  |  |  |
|  |  |  |  | *yfhL* | -1.1 |  | 10 |  |  |  |  |
|  |  |  |  | *secF* | -1.1 |  | 21 |  |  |  |  |
|  |  |  |  | *mrdA* | -1.1 |  | 34 |  |  |  |  |
|  |  |  |  | *trmA* | -1.1 |  | 44 |  |  |  |  |
|  |  |  |  | *hflD* | -1.1 |  | 61 |  |  |  |  |
|  |  |  |  | *ybiT* | -1.1 |  | 18 |  |  |  |  |
|  |  |  |  | *rplE* | -1.1 |  | 88 |  |  |  |  |
|  |  |  |  | *ndk* | -1.1 |  | 88 |  |  |  |  |
|  |  |  |  | *cdsA* | -1.1 |  | 38 |  |  |  |  |
|  |  |  |  | *pth* | -1.1 |  | 35 |  |  |  |  |
|  |  |  |  | *tsx* | -1.1 | + | 39 |  |  |  |  |
|  |  |  |  | *lpxD* | -1.1 |  | 35 |  |  |  |  |
|  |  |  |  | *fabH* | -1.1 |  | 23 |  |  |  |  |
|  |  |  |  | *yfgL* | -1.1 |  | 30 |  |  |  |  |
|  |  |  |  | *yliH* | -1.1 |  | 35 |  |  |  |  |
|  |  |  |  | *ansA* | -1.1 |  | 44 |  |  |  |  |
|  |  |  |  | *msbA* | -1.1 |  | 24 |  |  |  |  |
|  |  |  |  | *sscR* | -1.1 |  | 47 |  |  |  |  |
|  |  |  |  | *aroK* | -1.1 |  | 31 |  |  |  |  |
|  |  |  |  | *rfbB* | -1.1 |  | 34 |  |  |  |  |
|  |  |  |  | *rnhB* | -1.1 |  | 38 |  |  |  |  |
|  |  |  |  | *rfaQ* | -1.1 |  | 13 |  |  |  |  |
|  |  |  |  | *ilvN* | -1.0 |  |  |  |  |  |  |
|  |  |  |  | *tolR* | -1.0 |  | 25 |  |  |  |  |
|  |  |  |  | *trmI* | -1.0 | + | 33 |  |  |  |  |
|  |  |  |  | *artQ* | -1.0 |  | 86 |  |  |  |  |
|  |  |  |  | *ybeA* | -1.0 |  | 27 |  |  |  |  |
|  |  |  |  | *secD* | -1.0 | + | 20 |  |  |  |  |
|  |  |  |  | *maa* | -1.0 | + | 75 |  |  |  |  |
|  |  |  |  | *yciA* | -1.0 |  | 37 |  |  |  |  |
|  |  |  |  | *alaW* | -1.0 |  | 69 |  |  |  |  |
|  |  |  |  | *alaX* | -1.0 |  | 73 |  |  |  |  |
|  |  |  |  | *metN* | -1.0 |  | 27 |  |  |  |  |
|  |  |  |  | *yejL* | -1.0 |  | 37 |  |  |  |  |
|  |  |  |  | *upp* | -1.0 |  | 48 |  |  |  |  |

^a^ Values shown represent the log_2_ ratio of expression indices compared to those at time zero. Expression ratios of 2-fold or greater are shown. Percent recovery is defined under Results; a blank cell indicates recovery could not be defined.

^b^Ref [11]
